# The Optimal Selection of Mother Wavelet Function and Decomposition Level for Denoising of DCG Signal

**DOI:** 10.3390/s21051851

**Published:** 2021-03-06

**Authors:** Young In Jang, Jae Young Sim, Jong-Ryul Yang, Nam Kyu Kwon

**Affiliations:** Department of Electronic Engineering, Yeungnam University, Gyeongsan, Gyeongbuk 38541, Korea; owlisie@yu.ac.kr (Y.I.J.); ja2922@yu.ac.kr (J.Y.S.)

**Keywords:** doppler cardiogram, wavelet transform, denoising, mother wavelet function, decomposition level, signal decomposition, signal-to-noise-ratio

## Abstract

The aim of this paper is to find the optimal mother wavelet function and wavelet decomposition level when denoising the Doppler cardiogram (DCG), the heart signal obtained by the Doppler radar sensor system. To select the best suited mother wavelet function and wavelet decomposition level, this paper presents the quantitative analysis results. Both the optimal mother wavelet and decomposition level are selected by evaluating signal-to-noise-ratio (SNR) efficiency of the denoised signals obtained by using the wavelet thresholding method. A total of 115 potential functions from six wavelet families were examined for the selection of the optimal mother wavelet function and 10 levels (1 to 10) were evaluated for the choice of the best decomposition level. According to the experimental results, the most efficient selections of the mother wavelet function are “db9” and “sym9” from Daubechies and Symlets families, and the most suitable decomposition level for the used signal is seven. As the evaluation criterion in this study rates the efficiency of the denoising process, it was found that a mother wavelet function longer than 22 is excessive. The experiment also revealed that the decomposition level can be predictable based on the frequency features of the DCG signal. The proposed selection of the mother wavelet function and the decomposition level could reduce noise effectively so as to improve the quality of the DCG signal in information field.

## 1. Introduction

Cardiovascular diseases (CVDs) are the leading and rapidly increasing cause of death in modern society. They caused 32.1% of annual global deaths in 2015 and the occurrence is growing [1]. According to this trend, research on heartbeat detection and heart rate variability analysis has been gaining increasing attention. As the basic method of the heart signal recording, the electrocardiogram (ECG) test is used for measuring the electrophysiological signal of the heart activity to diagnose symptoms of cardiovascular problems [2]. The ECG test can improve the diagnosis and therapy of the most prevalent cardiac diseases if it does not interfere with daily activities [3]. However, some requirements of the ECG test make subjects uncomfortable. For example, subjects are required to attach multiple electrodes on their skin and remain in static state for quite some time [4]. In addition as the abnormal heartbeat signal appears for a very short time, the ECG test could miss these short and temporary symptoms because this test only records in limited circumstances.

Doppler radar sensors detect the electrophysiological signal of the heart and the variation of blood vessel movement by differences of the electromagnetic signal that is a few dozens of centimeters away from a subject [5,6,7,8]. Due to the non-contactable and flexible measurement condition, as opposed to ECG sensing, the Doppler radar sensors are studied for the alternative of ECG electrodes [9]. However, the noise occurs in the output signal obtained by the radar sensors due to the limitation of the non-contact measurement. Since the noise has a negative effect on data acquisition and processing, it leads to a decrease in information accuracy. Therefore, the method for signal denoising is essential and a considerable amount of research related to denoising has been studied [10,11,12,13,14]. Among them, the noise reduction methods using the wavelet analysis are widely studied not only for the radar signal but also for various multi-dimensional signals [15,16,17,18,19,20].

Noise reduction methods using the wavelet transform and thresholding can improve the quality of the Doppler cardiogram (DCG) and overcome the limits of remote sensors. To successfully enhance signal quality, the noise reduction technique should satisfy the performance of removing the noise components and maintaining useful signal components. In comparison to other noise removal methods such as average filtering or frequency smoothing using the fast Fourier transform, the wavelet denoising method shows a superior performance for preserving the features of the original signal when removing noise components. In particular, it is effective in regards to the problem of non-stationary signals such as a heart signal, respiration signal, and ocular artifacts [21,22,23,24,25]. The multiresolution decomposition of the wavelet transform makes it possible to maintain the original information during the process of removing noise components. Meanwhile, since various scaling factors and features of the wavelet functions are available, the optimal selection procedure of the mother wavelet function is a significant step to the wavelet denoising method [26,27]. Traditionally, the mother wavelet function is selected to represent the characteristics of the signal by empirical research [28,29]. Since this method does not provide the actual optimal selection of the mother wavelet [30], studies of the mother wavelet selection for various signals such as the electroencephalography (EEG) signal, vibration signal of turbine, human voice signal, and many kinds of radar signal have been occurring [26,28,31,32,33,34,35,36]. However, to the best of the author’s knowledge, studies on the optimal selection of the mother wavelet function and the decomposition level for the denoising of the DCG signal are rarely reported. Though selection of the mother wavelet function and the decomposition level on other radar signals were presented [35,36], distinctive features of each kind of radar signal require an individualized selection respectively, which is the motivation behind this study.

In this study, the optimal selection of the mother wavelet function and the decomposition level for the cardiac signal obtained by the Doppler radar sensors is proposed with quantitative analysis results. The result of the denoising process is defined with the signal-to-noise-ratio (SNR) efficiency of the denoised signal. The candidates for the optimal mother wavelet function are composed of 115 functions from six different wavelet families and the decomposition level has 10 candidates from 1 to 10. To determine the optimal mother wavelet function and the optimal decomposition level, the denoising process is repeated to examine all candidates and then all results are accumulated. The optimal choice of the mother wavelet function and the decomposition level is selected among the accumulated results which perform the highest SNR efficiency. The experiment is executed in two steps to find out the optimal selection of the mother wavelet function and the decomposition level. For the first step, the optimal mother wavelet function is determined by using the arbitrary wavelet decomposition level. Then the optimal decomposition level is obtained by using the optimal mother wavelet function which is selected at the first step. In this study, the arbitrary decomposition level in the first step is predicted based on the distribution of the signal components in the frequency domain. With these experiments, it is found that the length of the mother wavelet function is one of the key determinants for the optimal selection. The noisy signals were generated by adding white Gaussian noise to the original signals. The DCG dataset has 28 recordings that have been recorded for 160 s with a sampling frequency of 1000 Hz.

From the experimental results, it can be seen that the optimal mother wavelet function is found as db9 and sym9, and the optimal wavelet decomposition level is seven, respectively. The main contributions of this study can be described as follows:

- The optimal selection of the mother wavelet function and the decomposition level for the DCG (Doppler cardiogram) is introduced in this study;
- The optimal wavelet decomposition level is predicted based on the distribution of the signal components in frequency domain;
- The new criterion is suggested to evaluate both the denoising performance and the denoising efficiency in once.

## 2. Materials and Methods

### 2.1. Wavelet Transform

**Figure 1:**
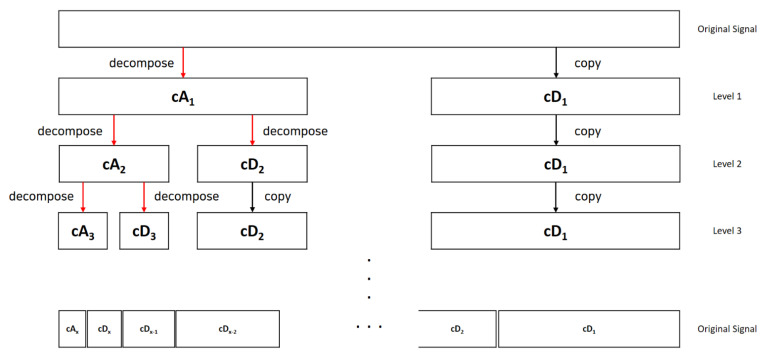
Illustration of the x level decomposition process.

The wavelet transform, a linear transformation with the mother wavelet function, is an efficient signal analysis method for both time and frequency resolution [22,23,37]. The wavelet transform has advantages over traditional Fourier transforms for accurately deconstructing and reconstructing finite, non-periodic, and non-stationary signals such as DCG signals. The wavelet transform decomposes the input signal into detail coefficients (cD) and approximation coefficients (cA) that can be defined as high frequency coefficients ($y_{high}\left[n\right]$) and low frequency coefficients ($y_{low}\left[n\right]$), respectively [38]. The frequency band coefficients of the wavelet transform are expressed mathematically as follows [38]:

(1) $$y_{high}\left[n\right]=\overset{\infty }{\underset{i=\;-\infty }{\sum }}s\left[i\right]h\left[2n-i\right],$$

(2) $$y_{low}\left[n\right]=\overset{\infty }{\underset{i=\;-\infty }{\sum }}s\left[i\right]g\left[2n-i\right],$$

where i is a sampling data point; n is the number of the sampling data; s[i] is the discrete radar signal with noise; and g[2n−i] and h[2n−i] are low-pass and high-pass filters that vary depending on the mother wavelet function [39]. With frequency filters, the wavelet transform enables one to extract the particular frequency band from the original signal [40,41]. Figure 1 shows the x level decomposition process into the approximation coefficients cA and the detail coefficients cD of the N Hz signal:

The wavelet function is composed with the scaled and translated copies of the scaling function ϕ(x) and the mother wavelet function ψ(x) [26,38] which is a continuously differentiable function with compact support. Since there are many different types of mother wavelet functions, finding the optimal mother wavelet is essential for the best performance not only in denoising but also in other signal processing. The coefficients of the discrete wavelet transform represent the projection of the signal over a set of basis functions generated as the translation and dilation of the mother wavelet function and the scaling function [42]. More specifically, the low-pass coefficients are related to the scaling function and the high-pass coefficients are related to the mother wavelet function as in Equations (3)–(5) [38]:

(3) $$g\left(h\right)={\left(-1\right)}^{n}h\left(1-n\right),$$

(4) $$\phi \left(x\right)=\;\sum_{n}h\left(n\right)\sqrt{2}\phi \left(2x-n\right),$$

(5) $$\psi \left(x\right)=\;\sum_{n}g\left(n\right)\sqrt{2}\phi \left(2x-n\right).$$

The low-pass and high-pass filters are distinctive variables for each mother wavelet function. Since the selection of the mother wavelet function and the decomposition level affects the noise reduction performance, the suitable mother wavelet function and the decomposition level could be optimizing the performance of the processing methods based on the wavelet transform.

### 2.2. Doppler Cardiogram Signal Dataset and Recording Procedure

A total of 28 DCG signals from 11 subjects were examined in this study. The datasets were recorded from healthy control subjects composed of 6 females and 5 males aged 24.08 ± 2.35 years (mean ± standard deviation). The subjects were recruited from the Electronic Engineering department of Yeungnam University. The subjects have no history of CVDs. Each signal data was recorded for 160 s and 1 k samples per second. The measurement system was constructed with a Doppler radar sensor, which measured the cardiogram of the subject at a distance of 30 cm. Table 1 shows the socio-demographic and clinical data of the control subjects. Permission to use the DCG data was granted from all subjects.

**Figure 2:**
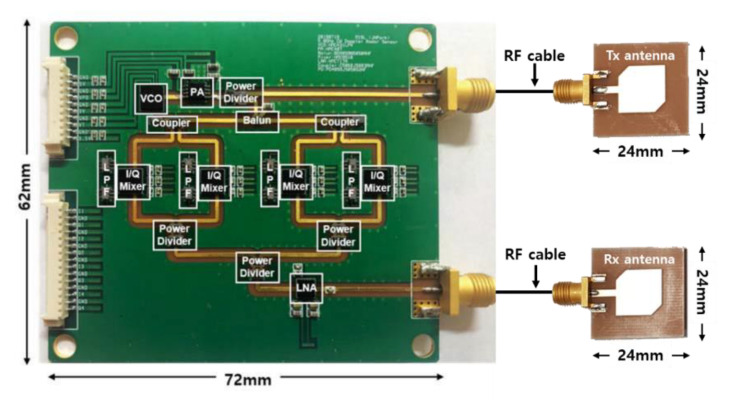
A 5.8 GHz continuous-wave Doppler radar sensors for the measurement of vital signs [43].

The DCG signals were detected by using the radar sensor module with two discrete patch antennas, which was implemented on an FR4 printed circuit board with a thickness of 0.6 mm, as shown in Figure 2 [43]. A total of 5.8 GHz of signals were generated by a voltage-controlled oscillator (HMC431LP4, Analog Devices) with an output power of 7.8 dBm radiating from the transmitting antenna with a directivity of 4.0 dBi. The received signals modulated by respiration and heartbeat were incident by using the receiving antenna, and they are down-converted to baseband signals by using quadrature mixers with low-pass filters with a cut-off frequency of 500 MHz. The overall noise figure in the receiver path was calculated to approximately 1.9 dB. The baseband signals were collected on a PC by using the data acquisition board with a sampling frequency of 1 kHz. Vital signs were obtained by filtering the baseband signals with a high-order band-pass filter in the digital domain. The multi-phase mixing in the radar module was used in the DC offset compensation in the received signals to improve the accuracy of the phase demodulation in signal processing [43].

**Figure 3:**
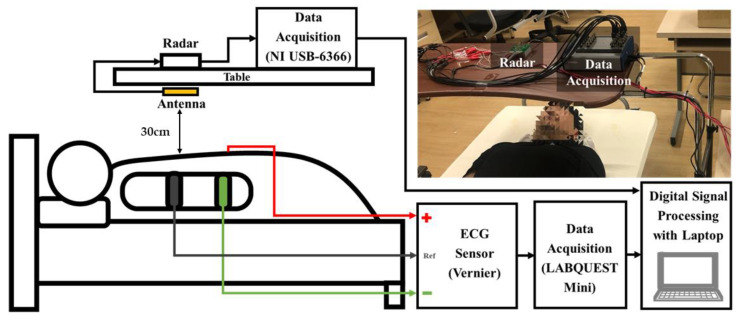
Block diagram of the experimental setup for vital signal detection using the 5.8 GHz CW radar.

Figure 3 shows the experimental setup for vital signs detection with a 5.8 GHz CW Doppler radar. The vital signs detected by the radar, which is located at a distance of 30 cm from the thorax of the subject and fixed on the bottom of the table above the subject, were obtained by using the data acquisition board (NI USB-6366, National Instruments, Austin, TX, USA) at a sampling rate of 1 kHz. A three-electrode ECG sensor (Vernier Software and Technology, Beaverton, OR, USA.), which was used as the reference sensor for heartbeat detection of the radar, was obtained at a sampling rate of 200 Hz. Heartbeat signals simultaneously obtained by the radar and ECG sensors were compared using NI LabVIEW and MATLAB on a computer. The vital signals of 11 subjects (5 males and 6 females in their 20 s) lying on the bed were continuously measured for 160 s. The movement of the subjects were limited to minimize the effect of the noise generated by motion on the ECG and radar sensors.

Though the ECG signals were not used in this study, ECG and DCG signals were measured from each subject once due to the structure of the sensor system. The corresponding matches of the ECG and DCG were obtained as a dataset for the future work of cardiogram analysis.

### 2.3. Evalution Measures

In this section, the optimal choice will be derived based on the quantitative experimental results. Since the SNR is one of the measures that evaluates the denoising performance [44,45], the following performance indexes were used to evaluate the denoised signal with j and k.

(6) $$SNR_{j,\;k}=10{log}_{10}\frac{\sum_{i=1}^{N}s^{2}\left[i\right]}{\sum_{i=1}^{N}\left|s\left[i\right]\;-\;{\hat{s}}_{j,\;k}\left[i\right]\right|},$$

(7) $$\eta \left(k\right)={log}_{10}\left(\left(s_{l}\;-\;l\left(m\left[k\right]\right)\right)\times l\left(m\left[k\right]\right)\right),$$

(8) $$eff\left(j,\;k\right)=\frac{SNR_{j,k}}{\eta \left(k\right)}$$

where ${\hat{s}}_{j,k}\left[i\right]$ and $SNR_{j,\;k}$ in (10) denote the denoised signal and the SNR when the decomposed level is j and the wavelet function index is k. The η(k) (11) is the execution complexity of the wavelet transform in case the wavelet function index is k. The η(k) is defined as the common logarithm value of the number of multiplication operations required for the wavelet transform that can be obtained by the length of the signal $s_{l}$ and the length of the mother wavelet function l(m[k]). For the denoising process, the higher SNR value shows that a better performance and lower execution complexity represents higher efficiency [46]. Therefore, the new criterion eff(j, k) (12) is proposed in this study to evaluate denoising performance efficiency.

## 3. Experiment

### 3.1. Additive White Gaussian Noise

**Figure 4:**
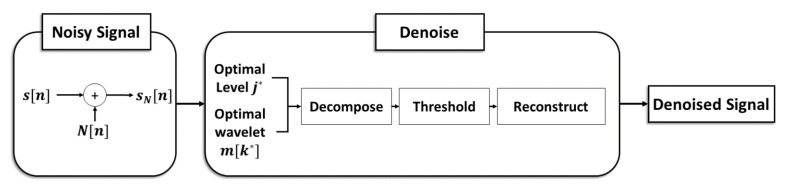
The block diagram of the denoising method.

Additive white Gaussian noise is the fundamental noise model in the information signal processing with three important characteristics manifested in its term. First, additive means the way in which noise joins in the signal. The noisy signal $s_{N}\left[n\right]$ is generated by adding the noise components N[n] to the original signal s[n] [47]. This process is described in Figure 4 and the mathematical expression is described as below:

(9) $$s_{N}\left[n\right]=s\left[n\right]+N\left[n\right].$$

Second, white denotes the power spectrum density of the noise is a constant value across the frequency range and the noise exists at almost every frequency [48]. Finally, Gaussian represents that the distribution of the noise is the Gaussian random process. The additive white Gaussian noise consists of normal distribution in the time domain and independent and identical distribution (i.i.d) in the frequency domain [49]. In this study, the additive white Gaussian noise components follow the distribution of N(0, σ), which is copied to the electromagnetic noise distribution of the original signal. Since the distribution is identical, the generated noisy signal $s_{N}\left[n\right]$ can represent comparable features with the real world noisy signal [50]. In the experiment, the white Gaussian noise is added to each original DCG signal with a SNR of 10 dB by the signal processing tool that is provided from Mathworks MATLAB.

### 3.2. Denoising Process with Wavelet Transform and Thresholding

To improve the quality of the noisy signal, the denoising process should hold the real data factors and remove the additive noise components [27]. In the previous studies, the noise reduction methods are generally based on the model simulation or spectral analysis, in which it is difficult to keep the complicated features of the signal [27,51,52,53]. On the other hand, the wavelet decomposition thresholding method can control the noisy signal to maintain the original signal components and separate the noise components more precisely. In this study, the denoising method is composed of three steps as follows [54,55]:

1. Wavelet Decomposition
  Select the decomposition level j and the mother wavelet function m[k]. Produce the wavelet coefficients through discrete wavelet transform with these two factors.
2. Thresholding
  Set the threshold value, which is calculated by the wavelet coefficients. Threshold the decomposed wavelet coefficients.
3. Wavelet Reconstruction
  Reconstruct the wavelet coefficients after thresholding using j and m[k].

Figure 4 describes the whole denoising process as a block diagram with an illustration of the three steps of the denoising method. j denotes the decomposition level of 1 to 10, m[k] is the mother wavelet function, and k is the function index from 1 to 115. Meanwhile, at the thresholding step, the soft thresholding is used for its superior thresholding performance to the hard thresholding [56]. There are various threshold methods for noise elimination such as Empirical Bayes [57], Block James–Stein [58], False Discovery Rate [59], Minimax Estimation [60], Stein’s Unbiased Risk Estimate [61], and Universal Threshold [62,63]. As the threshold method affects the denoising performance, studies on the powerful threshold determination method are kept reported [64,65]. In this study, the Universal Threshold is selected for its simple operation and powerful performance. The mathematical expressions for the soft thresholding are described in Equations (10)–(12) [51]:

(10) $$y_{s}\left(c\right)=\;\left\{\begin{array}{c} sgn\left(c\right)\cdot \left(\left|c\right|-T_{U}\right),\\ 0, \end{array}\;\begin{array}{c} \left|c\right|>\;T_{U}\\ \left|c\right|\leq \;T_{U} \end{array}\right\},$$

(11) $$T_{U}=\;\hat{\sigma }\sqrt{2log\left(N\right)},$$

(12) $$\hat{\sigma }=MAD/0.6745,$$

where c is the wavelet coefficient of the decomposed noisy signal [65,66] and $T_{U}$ is the universal threshold value proposed by Donoho and Johnstone [51,67]. N is the number of samples in the signal and MAD is the median absolute deviation of the wavelet coefficients. The meaning of $\hat{\sigma }$ is the estimate of the standard deviation of the noise. Following these equations, the threshold value is produced with the median value of the wavelet coefficients and the number of the sampling ratio in the signal.

### 3.3. The Process of the Optimal Selection of the Mother Wavelet Function and the Decomposition Level

**Figure 5:**
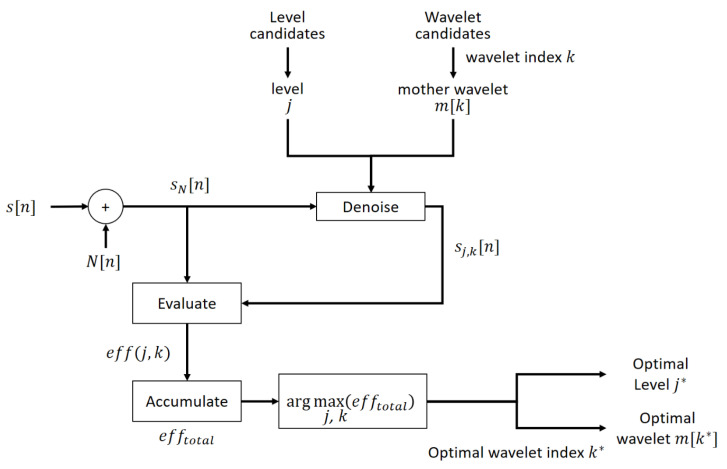
The block diagram of the optimal selection process for the mother wavelet function $m\left[k^{*}\right]$ and decomposition level $j^{*}$.

As the decomposition level and mother wavelet function have various candidates, the optimal selection not only evaluates a superior performance of denoising but also rates the execution efficiency to reduce unnecessary calculation. Table 2 and Figure 5 described the process of the optimal selection. First, the denoising process is repeated to accumulate the evaluation results for all candidates. Then, the optimal decomposition level and wavelet function index are obtained by finding the arguments of the maxima from the accumulated results. In Figure 5, $j^{*}$ denotes the optimal decomposition level and $m\left[k^{*}\right]$ denotes the optimal mother wavelet function, where $k^{*}$ is the function index of the optimal mother wavelet.

## 4. Result

### 4.1. Wavelet Decomposition Level Prediction

To determine the optimal mother wavelet function, the arbitrary decomposition level should be estimated. In this study, the decomposition level is estimated based on the dominant frequency range of the original signal. At the data analysis step, the original DCG signal has the dominant frequency band at 0 to 5 Hz. The approximation coefficients of the decomposition level seven obtain a frequency band of about 0 to 4 Hz, depending on the frequency decomposition rule [68]. Table 3, Table 4, Table 5, Table 6, Table 7 and Table 8 show the evaluation criterion eff(j, k) values for the candidates of the wavelet families at decomposition levels 3 to 8. According to Table 3, Table 4, Table 5, Table 6, Table 7 and Table 8, it can be shown that each wavelet family has the maximum eff(j, k) at seven. Thus, the estimated optimal decomposition level is set to seven.

### 4.2. Most Efficient Mother Wavelet Selection

**Figure 6:**
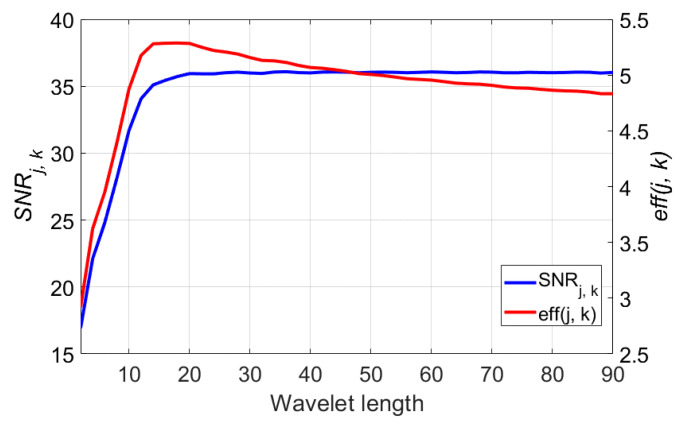
Denoising performance of the wavelet length (2 to 90), blue plot denotes $SNR_{j,\;k}$ values and red plot denotes eff(j, k). The used decomposition level j is 7.

In this section, the analytic results of the denoising performance using 115 wavelet functions are presented. The examined wavelet functions are selected from the six wavelet families including Coiflets (coif1-coif5), Daubechies (db1-db45), Fejer–Korovkin (fk4-fk22), Biorthogonal Spline (bior1.1-bior6.8), Reverse Biorthogonal Spline (rbio1.1-rbio6.8), and Symlets (sym2-sym30) [69,70]. The length of the wavelet varies by the number of the mother wavelet function name in range of 2 (db1, coif1, etc.) to 90 (db45), only even numbers. Traditionally, the optimal mother wavelet for noise reduction should satisfy the properties of orthogonality, symmetry, regularity, similarity of the shape with the signal, and so on [29,49,71]. According to the experiment result, however, the wavelet length of the mother wavelet function was the key determinant for the noise elimination of the DCG signal. The blue plot of the Figure 6 shows that the $SNR_{j,\;k}$ of the denoised signal increases with the wavelet length of the mother wavelet function and is saturated at a wavelet length of 18. Although the values of the $SNR_{j,\;k}$ from the wavelet length of 22 to 90 are similar, execution complexity keeping up with the wavelet length makes the longer wavelet function excessive. As the evaluation criterion eff(j, k) includes both the denoising performance $SNR_{j,\;k}$ and the execution complexity η(k), the most efficient wavelet length can be selected as 18 from the red plot of Figure 6. Not only does the execution efficiency become worse but the required length of the signal also increases when the wavelet length grows.

**Figure 7:**
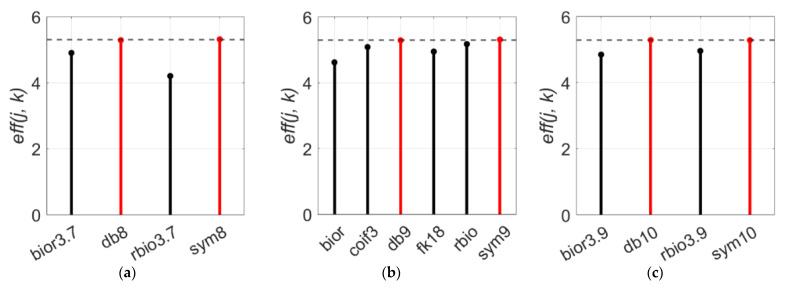
eff(j, k) values of six wavelet families (db, sym, coif, fk, bior, and rbio) for wavelet lengths of: (**a**) 16, (**b**) 18, and (**c**) 20. The used decomposition level j is 7.

However, the wavelet length is not the only feature that represents the characteristic of the mother wavelet function. Though few numbers of the wavelet functions share the same wavelet length, the basis functions and basic features are distinctive. Therefore, even with the same wavelet length, the denoising performance eff(j, k) will vary depending on the basis function of the mother wavelet function. At a wavelet length of 16, “db8” and “sym8” scored 5.28 and 5.31 which are higher than other wavelets and the average performance of a wavelet length of 16 is 4.92 (Figure 7a). In the case a wavelet length of 18, “db9” and “sym9” perform better again (Figure 7b). For a wavelet length of 20 (Figure 7c), “db“ and “sym” are always the optimal wavelet functions. On the contrary with the Daubechies and Symlets families, the function from the Biorthogonal family shows the lowest performance at all three graphs.

### 4.3. Optimal Decomposition Level

**Figure 8:**
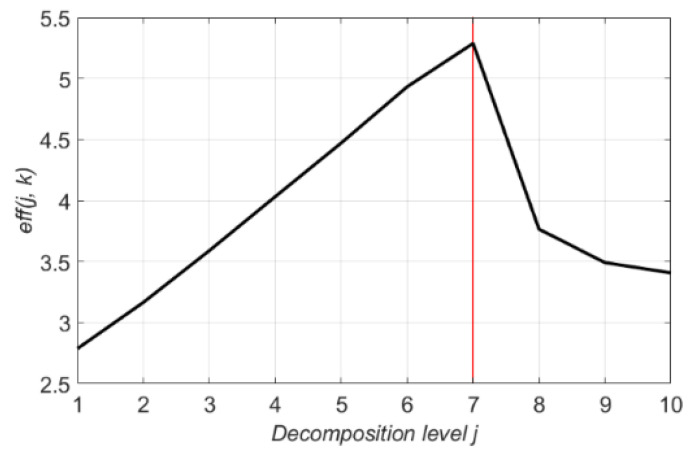
eff(j, k) values of 10 decomposition levels (1 to 10) for db9.

**Figure 9:**
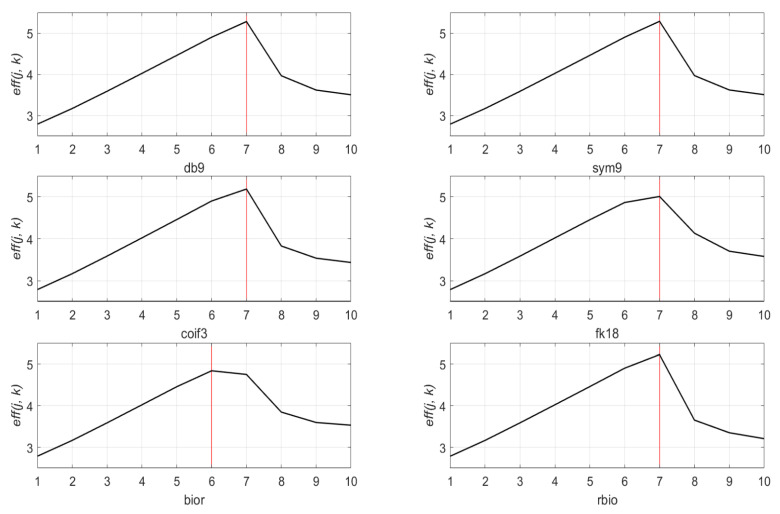
eff(j, k) values of 10 decomposition levels (1 to 10) for six mother wavelet functions (db9, sym9, coif3, fk18, bior, and rbio).

To optimize the performance of the denoising method using the wavelet transform and thresholding, determining the proper decomposition level is also necessary. However, the optimal decomposition level was first estimated in Section 4.1 based on the dominant frequency range and the sampling ratio of the signal. In this subsection, we will cover the results of the quantitative analysis for the optimal decomposition level. Figure 8 represents eff(j, k) of the denoised signal. The decomposition level in range from 1 to 10 is denoted as the x-axis, the eff(j, k) from the optimal mother wavelet “db9” is indicated as the y-axis. Figure 9 shows the eff(j, k) of the six wavelet families at decomposition level 1 to 10. Each wavelet function is fixed to a wavelet length of 18, which is the most efficient wavelet length obtained in the previous subsection. Through the experimental result analysis of Figure 8 and Figure 9, the denoising process with decomposition level seven achieves the best performance among all candidates but a higher decomposition level apparently gives less eff(j, k) performance. Although Figure 9 shows that the “bior” graph has a maximum value at level six, still the maximum eff(j, k) of the “bior” family is far less than the maximum eff(j, k) of other families, the wavelet functions from the “bior” family are out of consideration in this study. This incident happens when the wavelet length of the mother wavelet function is too short for the length of the input signal.

**Figure 10:**
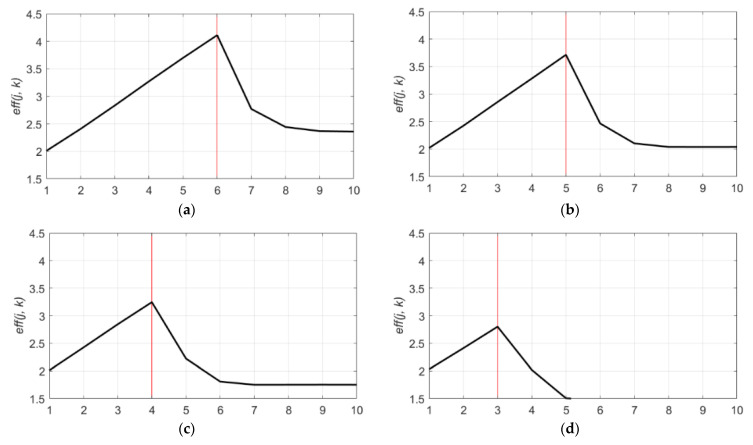
eff(j, k) values of 10 decomposition levels (1 to 10) for db9. (**a**) eff(j, k) values for a sampling frequency of 500 Hz; (**b**) eff(j, k) value for a sampling frequency of 250 Hz; (**c**) eff(j, k) values for a sampling frequency of 125 Hz; and (**d**) eff(j, k) values for a sampling frequency of 62 Hz.

**Figure 11:**
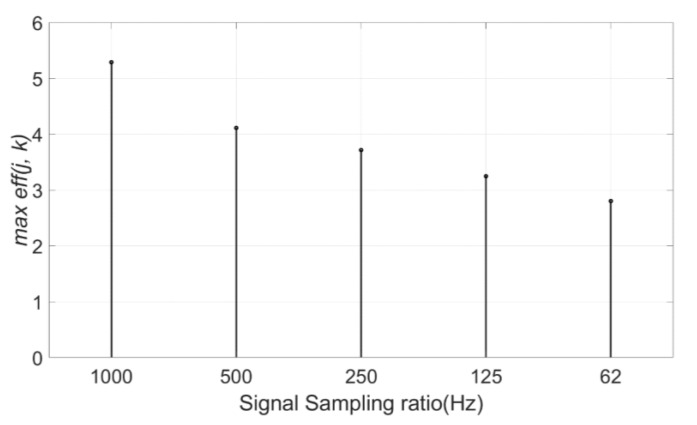
Maximum eff(j, k) values of signals with five sampling ratios (1000 Hz, 500 Hz, 250 Hz, 125 Hz, and 62 Hz).

The approximation coefficients at the optimal decomposition level is $cA_{7}$, which are composed of a partial signal whose frequency range is approximately 0~4 Hz. This frequency range is the subset of the dominant frequency band (0~5 Hz) of the original signal. Therefore, the decomposition level, which can extract the dominant frequency band of the original signal at approximation coefficients, is the optimal decomposition level for signal denoising. To prove this, the same denoising process was performed to the DCG signals with different sampling ratios. The DCG signals with a sampling frequency of 1000 Hz were sampled to different sampling ratios to provide identical DCG characteristics but different frequency features. The eff(j, k) values of these sampled signals with four different sampling ratios (500 Hz, 250 Hz, 125 Hz, and 62 Hz) are described in Figure 10. As shown in Figure 10a, in case of a sampling ratio of 500 Hz, the most effective decomposition level lowered 1 step to level 6 as the sampling ratio decreased to half of the original. At this time, the denoising performance also decreased. Likewise, the plot of the signal with the sampling frequency of 125 Hz in Figure 10c represents that the optimal decomposition level is four, which is three levels lower than the original. Moreover, the maximum eff(j, k) of the denoised signal decreases as the sampling ratio falls off (Figure 11). To summarize, the optimal decomposition level is determined by the dominant frequency band and the sampling ratio of the original signal. It is also shown that the sampling frequency of the signal affects to the denoising performance because the higher sampling rate can obtain more useful information components than the signal with a lower sampling rate.

## 5. Discussions

In this study, the denoising process using wavelet decomposition and thresholding method is performed to improve the quality of the DCG signal. Although there are many studies of the denoising process using the wavelet function [35,72,73,74], to the best of the author’s knowledge, previous studies have yet to provide the optimal mother wavelet selection for the DCG signal. The purpose of this study is proposing the optimal selection of the mother wavelet function and the decomposition level for the DCG signal to optimize the performance of the denoising process.

Xu et al. [35] and Srivastava et al. [36] proposed the selection of the mother wavelet function and the decomposition level of the object radar signal, respectively. However the proposed mother wavelet function and decomposition level for their object radar signal performed less powerfully on the DCG signal than the proposed selection from this study (Table 9). As a consequence, the optimal mother wavelet function and the decomposition level should be selected identically by analyzing the characteristics of the object signal.

The denoising process with the optimal selection for the DCG signal enhanced the quality of the DCG signals in the information field. As the DCG signals can be obtained in a more flexible condition than the ECG signals, the improvement of the DCG signals could enhance the diagnosis of the CVDs.

## 6. Conclusions

The wavelet decomposition thresholding is a powerful denoising method. The performance of the denoising method can be optimized by using the optimal set of the mother wavelet function and the decomposition level. For the DCG signal, this study suggested the optimal selection of the mother wavelet function and the decomposition level based on the signal analysis. To select the optimal mother wavelet function, the wavelet length of the mother wavelet function is an important element. In this study, the length of the examined signal was 160,000, with a 1000 Hz sampling rate signal recorded for 160 s and the most efficient wavelet length was 18. There are six wavelet families with wavelet length 18; “db9”, “sym9”, “coif3”, “fk18”, “bior”, and “rbio”. Most of these six functions recorded superior performance to other functions with a different wavelet length and “db9” and “sym9” were selected for the optimal mother wavelet functions among all 115 wavelet candidates. The optimal decomposition level for the DCG signal was determined as seven, which shows that the estimation of the decomposition level based on the length of the signal was precise.

Since the noise reduction method based on the wavelet decomposition and the thresholding with optimal parameters successfully removes the noise from the DCG signal, the quality of the heart rate signal obtained by Doppler radar sensors was improved in the information field. Therefore, analyzing a DCG signal using artificial neural networks for the diagnosis of CVDs is conducted as the aim of the future work following to this study.

The major findings in this study is denoted as follows:

- The wavelet length of the mother wavelet function was the important element for the selection of the most efficient mother wavelet. The longer mother wavelet function did not provide a better denoising performance. As the longer wavelet function requires more performance complexity, the optimal wavelet length for the performance efficiency should be considered;
- The optimal decomposition level was determined by the sampling frequency and dominant frequency range of the original signal. The level that could decompose the dominant frequency range from the signal was the optimal decomposition level. For this reason, the optimal decomposition level could be predicted based on the signal analysis in the frequency domain. The appropriate decomposition level produced a modest threshold value for noise removal;
- The higher the sampling frequency of the DCG signal, the more powerful the performance of the denoising process. The higher sampling frequency enabled the signal to obtain more useful components.

## Figures and Tables

**Table 1:** Sociodemographic data of the control subjects (Age in years, mean ± standard deviation, SD).

Demographic and Clinical Features	Control
Number	11
Age	24.08 ± 2.35
Female/Male	6 F/5 M

**Table 2:** The table of algorithm step for the analysis process of the optimal selection.

Algorithm Step
Accumulate the denoising result using 115 mother wavelet functions and decomposition level 7 (prediction).
Find out the most efficient mother wavelet function $m[k^{*}]$.
Accumulate the denoising result using the optimal mother wavelet function $m[k^{*}]$ and 10 decomposition levels.
Find out the most efficient wavelet decomposition level $j^{*}$.
Set the optimal selection of the mother wavelet function $m[k^{*}]$ and the wavelet decomposition level $j^{*}$.

**Table 3:** The eff(j, k) value of the Coiflets family (wavelet length 18–30).

	3	4	5	6	7	8
**18**	3.5903	4.0369	4.4724	4.9091	5.0815	3.7178
**24**	3.5252	3.9538	4.3936	4.8243	5.1840	3.6898
**30**	3.4757	3.8981	4.3334	4.7615	5.1429	3.6670

**Table 4:** The eff(j, k) value of the Daubechies family (wavelet length 18–22).

	3	4	5	6	7	8
**18**	3.5902	4.0328	4.4719	4.9322	5.2873	3.7658
**20**	3.5671	4.0081	4.4414	4.8843	5.2838	3.7557
**22**	3.5439	3.9737	4.4199	4.8498	5.2496	3.7590

**Table 5:** The eff(j, k) value of the Fejer–Korovkin family (wavelet length 18–22).

	3	4	5	6	7	8
**18**	3.5910	4.0327	4.4659	4.8763	4.9468	3.8317
**22**	3.5438	3.9782	4.4147	4.8194	4.9721	3.8463

**Table 6:** The eff(j, k) value of the Biorthogonal Spline family (wavelet length 18–20).

	3	4	5	6	7	8
**18**	3.5894	4.0377	4.4704	4.9072	5.1879	3.7663
**20**	3.5521	3.9645	4.3828	4.7759	4.8445	3.8774

**Table 7:** The eff(j, k) value of the Reverse Biorthogonal Spline family (wavelet length 18–20).

	3	4	5	6	7	8
**18**	3.5867	4.0361	4.4688	4.9040	5.1384	3.4258
**20**	3.5602	3.9910	4.4374	4.8952	4.9568	3.0319

**Table 8:** The eff(j, k) value of the Symlets family (wavelet length 18–20).

	3	4	5	6	7	8
**18**	3.5914	4.0267	4.4733	4.9256	5.3099	3.7781
**20**	3.5657	4.0102	4.4428	4.8765	5.2783	3.7603
**22**	3.5450	3.9741	4.4176	4.8723	5.2523	3.7297

**Table 9:** The table for the comparison of the $SNR_{j,\;k}$ performance between the Xu et al., Srivastava et al., and proposed selection.

	Proposed Selection	Xu et al. Selection [35]	Srivastava et al. Selection [36]
$SNR_{j,k}$ **/dB**	35.726	27.191	33.153
**Wavelet**	db9	db4	coif 3
**level**	7	4	6

## Data Availability

The data are not publicly available due to the company security policy and personal protection of subjects.
